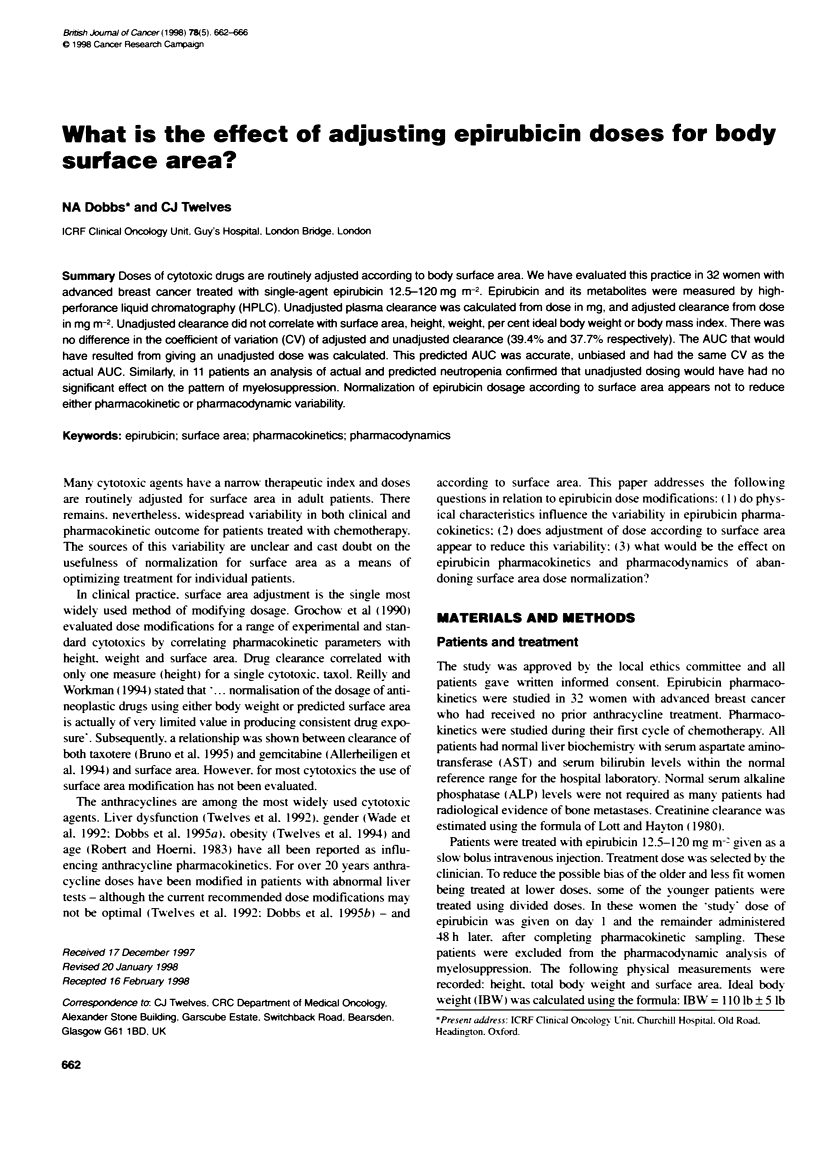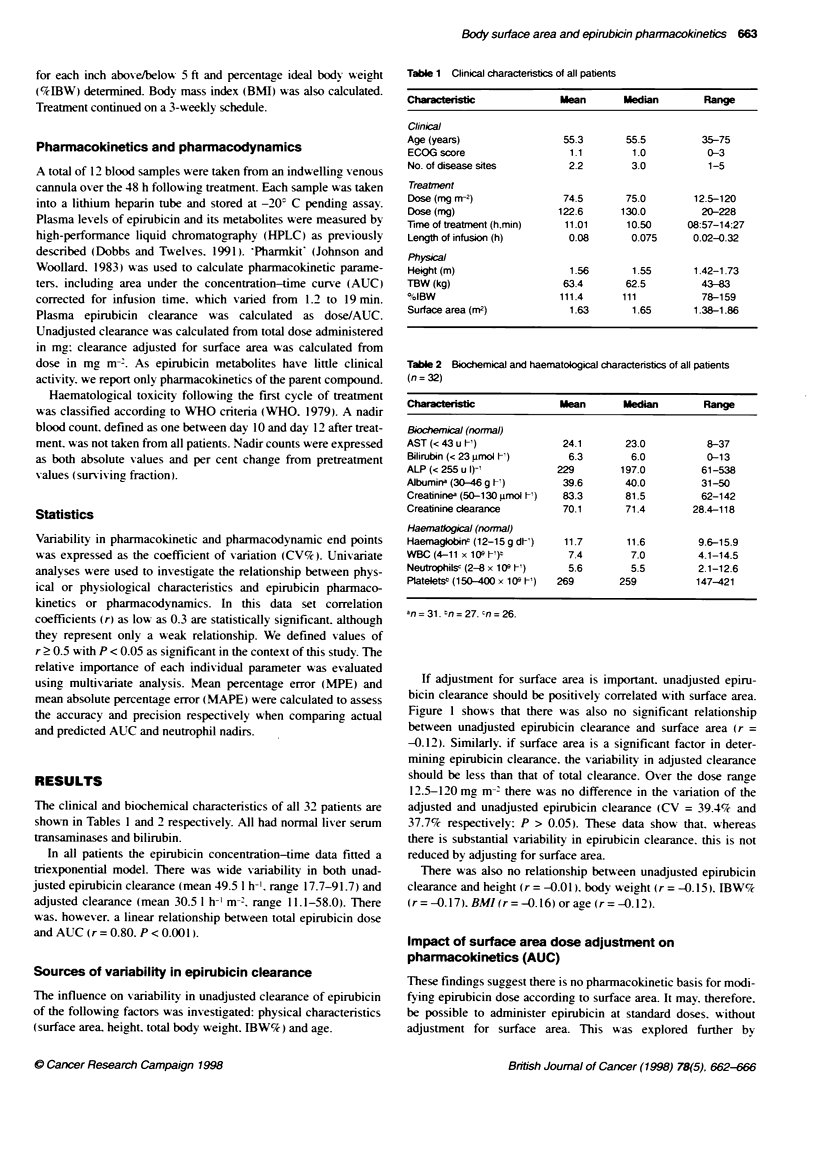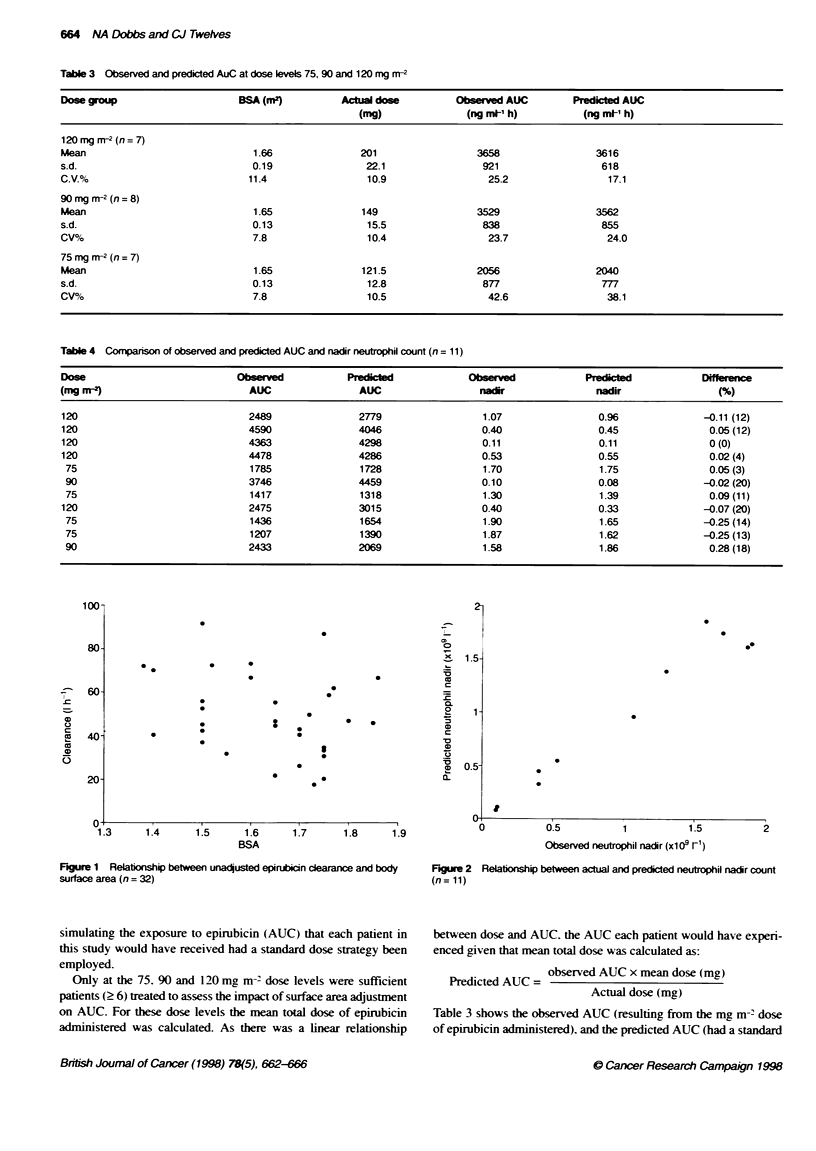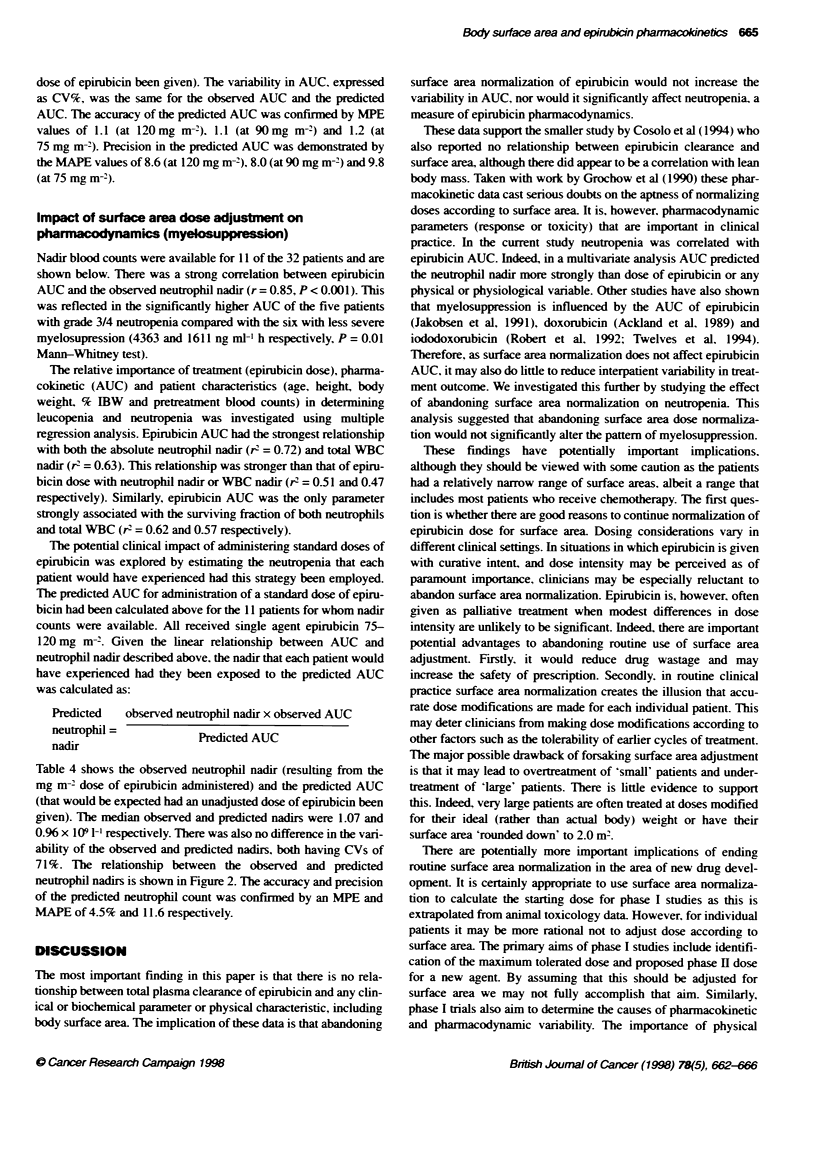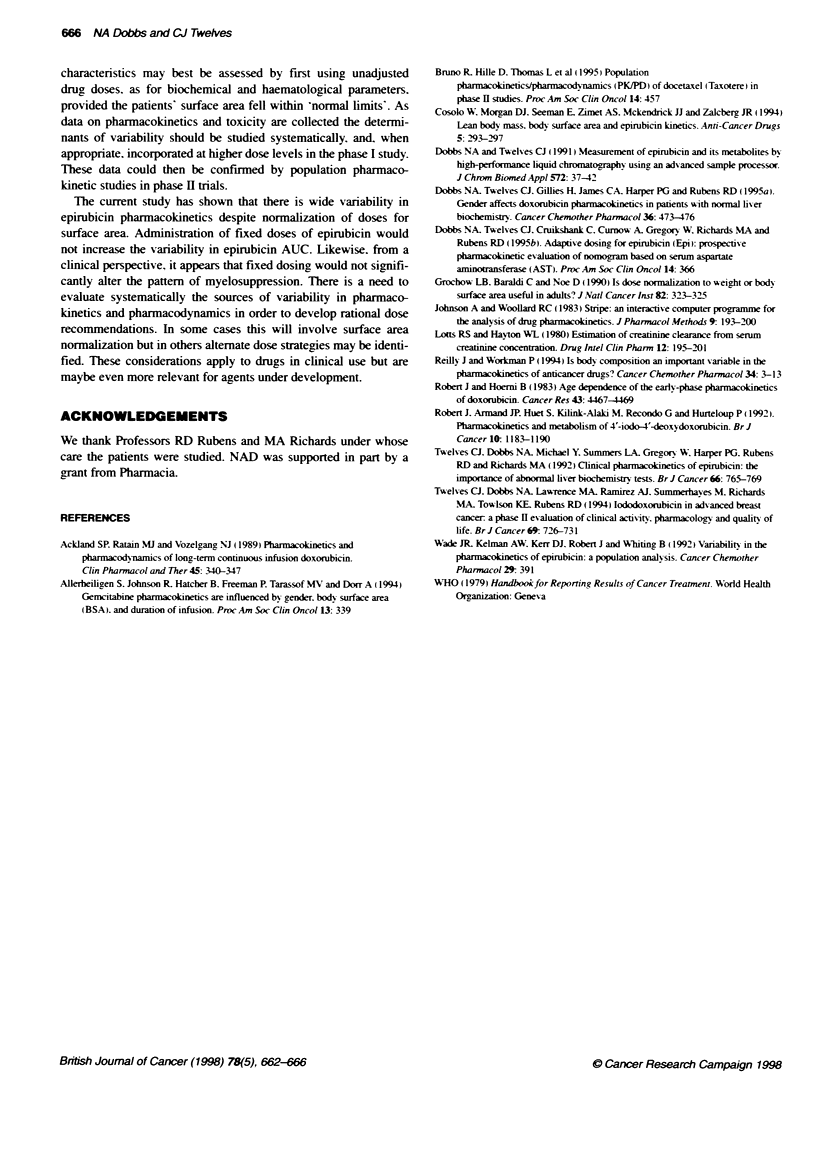# What is the effect of adjusting epirubicin doses for body surface area?

**DOI:** 10.1038/bjc.1998.556

**Published:** 1998-09

**Authors:** N. A. Dobbs, C. J. Twelves

**Affiliations:** ICRF Clinical Oncology Unit, Guy's Hospital, London.

## Abstract

Doses of cytotoxic drugs are routinely adjusted according to body surface area. We have evaluated this practice in 32 women with advanced breast cancer treated with single-agent epirubicin 12.5-120 mg m(-2). Epirubicin and its metabolites were measured by high-performance liquid chromatography (HPLC). Unadjusted plasma clearance was calculated from dose in mg, and adjusted clearance from dose in mg m(-2). Unadjusted clearance did not correlate with surface area, height, weight, per cent ideal body weight or body mass index. There was no difference in the coefficient of variation (CV) of adjusted and unadjusted clearance (39.4% and 37.7% respectively). The AUC that would have resulted from giving an unadjusted dose was calculated. This predicted AUC was accurate, unbiased and had the same CV as the actual AUC. Similarly, in 11 patients an analysis of actual and predicted neutropenia confirmed that unadjusted dosing would have had no significant effect on the pattern of myelosuppression. Normalization of epirubicin dosage according to surface area appears not to reduce either pharmacokinetic or pharmacodynamic variability.


British Journal of Cancer (1998) 78(5). 662–666
© 1998 Cancer Research Campaign

### What is the effect of adjusting epirubicin doses for body surface area?

**NA Dobbs*** **and CJ Twelves**

ICRF Clinical Oncology Unit. Guy's Hospital. London Bridge. London

**Summary** Doses of cytotoxic drugs are routinely adjusted according to body surface area. We have evaluated this practice in 32 women with advanced breast cancer treated with single-agent epirubicin 12.5–120 mg $m^{-2}$. Epirubicin and its metabolites were measured by high-perforance liquid chromatography (HPLC). Unadjusted plasma clearance was calculated from dose in mg, and adjusted clearance from dose in mg $m^{-2}$. Unadjusted clearance did not correlate with surface area, height, weight, per cent ideal body weight or body mass index. There was no difference in the coefficient of variation (CV) of adjusted and unadjusted clearance (39.4% and 37.7% respectively). The AUC that would have resulted from giving an unadjusted dose was calculated. This predicted AUC was accurate, unbiased and had the same CV as the actual AUC. Similarly, in 11 patients an analysis of actual and predicted neutropenia confirmed that unadjusted dosing would have had no significant effect on the pattern of myelosuppression. Normalization of epirubicin dosage according to surface area appears not to reduce either pharmacokinetic or pharmacodynamic variability.

**Keywords:** epirubicin; surface area; pharmacokinetics; pharmacodynamics

Many cytotoxic agents have a narrow therapeutic index and doses are routinely adjusted for surface area in adult patients. There remains, nevertheless, widespread variability in both clinical and pharmacokinetic outcome for patients treated with chemotherapy. The sources of this variability are unclear and cast doubt on the usefulness of normalization for surface area as a means of optimizing treatment for individual patients.

In clinical practice, surface area adjustment is the single most widely used method of modifying dosage. Grochow et al (1990) evaluated dose modifications for a range of experimental and standard cytotoxics by correlating pharmacokinetic parameters with height, weight and surface area. Drug clearance correlated with only one measure (height) for a single cytotoxic, taxol. Reilly and Workman (1994) stated that '... normalisation of the dosage of antineoplastic drugs using either body weight or predicted surface area is actually of very limited value in producing consistent drug exposure'. Subsequently, a relationship was shown between clearance of both taxotere (Bruno et al. 1995) and gemcitabine (Allerheiligen et al. 1994) and surface area. However, for most cytotoxics the use of surface area modification has not been evaluated.

The anthracyclines are among the most widely used cytotoxic agents. Liver dysfunction (Twelves et al. 1992), gender (Wade et al. 1992; Dobbs et al. 1995*a*), obesity (Twelves et al. 1994) and age (Robert and Hoerni. 1983) have all been reported as influencing anthracycline pharmacokinetics. For over 20 years anthracycline doses have been modified in patients with abnormal liver tests – although the current recommended dose modifications may not be optimal (Twelves et al. 1992; Dobbs et al. 1995*b*) – and according to surface area. This paper addresses the following questions in relation to epirubicin dose modifications: (1) do physical characteristics influence the variability in epirubicin pharmacokinetics; (2) does adjustment of dose according to surface area appear to reduce this variability; (3) what would be the effect on epirubicin pharmacokinetics and pharmacodynamics of abandoning surface area dose normalization?

### MATERIALS AND METHODS

### Patients and treatment

The study was approved by the local ethics committee and all patients gave written informed consent. Epirubicin pharmacokinetics were studied in 32 women with advanced breast cancer who had received no prior anthracycline treatment. Pharmacokinetics were studied during their first cycle of chemotherapy. All patients had normal liver biochemistry with serum aspartate aminotransferase (AST) and serum bilirubin levels within the normal reference range for the hospital laboratory. Normal serum alkaline phosphatase (ALP) levels were not required as many patients had radiological evidence of bone metastases. Creatinine clearance was estimated using the formula of Lott and Hayton (1980).

Patients were treated with epirubicin 12.5–120 mg $m^{-2}$ given as a slow bolus intravenous injection. Treatment dose was selected by the clinician. To reduce the possible bias of the older and less fit women being treated at lower doses, some of the younger patients were treated using divided doses. In these women the 'study' dose of epirubicin was given on day 1 and the remainder administered 48 h later, after completing pharmacokinetic sampling. These patients were excluded from the pharmacodynamic analysis of myelosuppression. The following physical measurements were recorded: height, total body weight and surface area. Ideal body weight (IBW) was calculated using the formula: IBW = 110 lb ± 5 lb

*Received 17 December 1997*
*Revised 20 January 1998*
*Recepted 16 February 1998*

*Correspondence to:* CJ Twelves. CRC Department of Medical Oncology. Alexander Stone Building, Garscube Estate, Switchback Road, Bearsden, Glasgow G61 1BD, UK

**Present address*: ICRF Clinical Oncology Unit. Churchill Hospital. Old Road. Headington. Oxford.

for each inch above/below 5 ft and percentage ideal body weight (%IBW) determined. Body mass index (BMI) was also calculated. Treatment continued on a 3-weekly schedule.

### Pharmacokinetics and pharmacodynamics

A total of 12 blood samples were taken from an indwelling venous cannula over the 48 h following treatment. Each sample was taken into a lithium heparin tube and stored at –20° C pending assay. Plasma levels of epirubicin and its metabolites were measured by high-performance liquid chromatography (HPLC) as previously described (Dobbs and Twelves, 1991). 'Pharmkit' (Johnson and Woollard, 1983) was used to calculate pharmacokinetic parameters, including area under the concentration–time curve (AUC) corrected for infusion time, which varied from 1.2 to 19 min. Plasma epirubicin clearance was calculated as dose/AUC. Unadjusted clearance was calculated from total dose administered in mg; clearance adjusted for surface area was calculated from dose in mg $m^{-2}$. As epirubicin metabolites have little clinical activity, we report only pharmacokinetics of the parent compound.

Haematological toxicity following the first cycle of treatment was classified according to WHO criteria (WHO, 1979). A nadir blood count, defined as one between day 10 and day 12 after treatment, was not taken from all patients. Nadir counts were expressed as both absolute values and per cent change from pretreatment values (surviving fraction).

### Statistics

Variability in pharmacokinetic and pharmacodynamic end points was expressed as the coefficient of variation (CV%). Univariate analyses were used to investigate the relationship between physical or physiological characteristics and epirubicin pharmacokinetics or pharmacodynamics. In this data set correlation coefficients ($r$) as low as 0.3 are statistically significant, although they represent only a weak relationship. We defined values of $r \geq 0.5$ with $P < 0.05$ as significant in the context of this study. The relative importance of each individual parameter was evaluated using multivariate analysis. Mean percentage error (MPE) and mean absolute percentage error (MAPE) were calculated to assess the accuracy and precision respectively when comparing actual and predicted AUC and neutrophil nadirs.

### RESULTS

The clinical and biochemical characteristics of all 32 patients are shown in Tables 1 and 2 respectively. All had normal liver serum transaminases and bilirubin.

In all patients the epirubicin concentration–time data fitted a triexponential model. There was wide variability in both unadjusted epirubicin clearance (mean 49.5 l $h^{-1}$, range 17.7–91.7) and adjusted clearance (mean 30.5 l $h^{-1}$ $m^{-2}$, range 11.1–58.0). There was, however, a linear relationship between total epirubicin dose and AUC ($r = 0.80$, $P < 0.001$).

### Sources of variability in epirubicin clearance

The influence on variability in unadjusted clearance of epirubicin of the following factors was investigated: physical characteristics (surface area, height, total body weight, IBW%) and age.

**Table 1** Clinical characteristics of all patients

| Characteristic | Mean | Median | Range |
|---|---|---|---|
| *Clinical* | | | |
| Age (years) | 55.3 | 55.5 | 35–75 |
| ECOG score | 1.1 | 1.0 | 0–3 |
| No. of disease sites | 2.2 | 3.0 | 1–5 |
| *Treatment* | | | |
| Dose (mg $m^{-2}$) | 74.5 | 75.0 | 12.5–120 |
| Dose (mg) | 122.6 | 130.0 | 20–228 |
| Time of treatment (h.min) | 11.01 | 10.50 | 08:57–14:27 |
| Length of infusion (h) | 0.08 | 0.075 | 0.02–0.32 |
| *Physical* | | | |
| Height (m) | 1.56 | 1.55 | 1.42–1.73 |
| TBW (kg) | 63.4 | 62.5 | 43–83 |
| %IBW | 111.4 | 111 | 78–159 |
| Surface area ($m^2$) | 1.63 | 1.65 | 1.38–1.86 |

**Table 2** Biochemical and haematological characteristics of all patients ($n$ = 32)

| Characteristic | Mean | Median | Range |
|---|---|---|---|
| *Biochemical (normal)* | | | |
| AST (< 43 u $l^{-1}$) | 24.1 | 23.0 | 8–37 |
| Bilirubin (< 23 μmol $l^{-1}$) | 6.3 | 6.0 | 0–13 |
| ALP (< 255 u $l)^{-1}$ | 229 | 197.0 | 61–538 |
| Albumin[a] (30–46 g $l^{-1}$) | 39.6 | 40.0 | 31–50 |
| Creatinine[a] (50–130 μmol $l^{-1}$) | 83.3 | 81.5 | 62–142 |
| Creatinine clearance | 70.1 | 71.4 | 28.4–118 |
| *Haematlogical (normal)* | | | |
| Haemaglobin[b] (12–15 g $dl^{-1}$) | 11.7 | 11.6 | 9.6–15.9 |
| WBC (4–11 × $10^9$ $l^{-1}$)[c] | 7.4 | 7.0 | 4.1–14.5 |
| Neutrophils[c] (2–8 × $10^9$ $l^{-1}$) | 5.6 | 5.5 | 2.1–12.6 |
| Platelets[b] (150–400 × $10^9$ $l^{-1}$) | 269 | 259 | 147–421 |

[a]$n$ = 31. [b]$n$ = 27. [c]$n$ = 26.

If adjustment for surface area is important, unadjusted epirubicin clearance should be positively correlated with surface area. Figure 1 shows that there was also no significant relationship between unadjusted epirubicin clearance and surface area ($r$ = –0.12). Similarly, if surface area is a significant factor in determining epirubicin clearance, the variability in adjusted clearance should be less than that of total clearance. Over the dose range 12.5–120 mg $m^{-2}$ there was no difference in the variation of the adjusted and unadjusted epirubicin clearance (CV = 39.4% and 37.7% respectively; $P > 0.05$). These data show that, whereas there is substantial variability in epirubicin clearance, this is not reduced by adjusting for surface area.

There was also no relationship between unadjusted epirubicin clearance and height ($r = -0.01$), body weight ($r = -0.15$), IBW% ($r = -0.17$), *BMI* ($r = -0.16$) or age ($r = -0.12$).

### Impact of surface area dose adjustment on pharmacokinetics (AUC)

These findings suggest there is no pharmacokinetic basis for modifying epirubicin dose according to surface area. It may, therefore, be possible to administer epirubicin at standard doses, without adjustment for surface area. This was explored further by

© Cancer Research Campaign 1998

**Table 3** Observed and predicted AuC at dose levels 75, 90 and 120 mg m$^{-2}$

| Dose group | BSA (m$^2$) | Actual dose (mg) | Observed AUC (ng ml$^{-1}$ h) | Predicted AUC (ng ml$^{-1}$ h) |
|---|---|---|---|---|
| 120 mg m$^{-2}$ ($n$ = 7) | | | | |
| Mean | 1.66 | 201 | 3658 | 3616 |
| s.d. | 0.19 | 22.1 | 921 | 618 |
| C.V.% | 11.4 | 10.9 | 25.2 | 17.1 |
| 90 mg m$^{-2}$ ($n$ = 8) | | | | |
| Mean | 1.65 | 149 | 3529 | 3562 |
| s.d. | 0.13 | 15.5 | 838 | 855 |
| CV% | 7.8 | 10.4 | 23.7 | 24.0 |
| 75 mg m$^{-2}$ ($n$ = 7) | | | | |
| Mean | 1.65 | 121.5 | 2056 | 2040 |
| s.d. | 0.13 | 12.8 | 877 | 777 |
| CV% | 7.8 | 10.5 | 42.6 | 38.1 |

**Table 4** Comparison of observed and predicted AUC and nadir neutrophil count ($n$ = 11)

| Dose (mg m$^{-2}$) | Observed AUC | Predicted AUC | Observed nadir | Predicted nadir | Difference (%) |
|---|---|---|---|---|---|
| 120 | 2489 | 2779 | 1.07 | 0.96 | –0.11 (12) |
| 120 | 4590 | 4046 | 0.40 | 0.45 | 0.05 (12) |
| 120 | 4363 | 4298 | 0.11 | 0.11 | 0 (0) |
| 120 | 4478 | 4286 | 0.53 | 0.55 | 0.02 (4) |
| 75 | 1785 | 1728 | 1.70 | 1.75 | 0.05 (3) |
| 90 | 3746 | 4459 | 0.10 | 0.08 | –0.02 (20) |
| 75 | 1417 | 1318 | 1.30 | 1.39 | 0.09 (11) |
| 120 | 2475 | 3015 | 0.40 | 0.33 | –0.07 (20) |
| 75 | 1436 | 1654 | 1.90 | 1.65 | –0.25 (14) |
| 75 | 1207 | 1390 | 1.87 | 1.62 | –0.25 (13) |
| 90 | 2433 | 2069 | 1.58 | 1.86 | 0.28 (18) |

**Figure 1** Relationship between unadjusted epirubicin clearance and body surface area ($n$ = 32)

**Figure 2** Relationship between actual and predicted neutrophil nadir count ($n$ = 11)

simulating the exposure to epirubicin (AUC) that each patient in this study would have received had a standard dose strategy been employed.

Only at the 75, 90 and 120 mg m$^{-2}$ dose levels were sufficient patients ($\geq$ 6) treated to assess the impact of surface area adjustment on AUC. For these dose levels the mean total dose of epirubicin administered was calculated. As there was a linear relationship between dose and AUC, the AUC each patient would have experienced given that mean total dose was calculated as:

$$\text{Predicted AUC} = \frac{\text{observed AUC} \times \text{mean dose (mg)}}{\text{Actual dose (mg)}}$$

Table 3 shows the observed AUC (resulting from the mg m$^{-2}$ dose of epirubicin administered), and the predicted AUC (had a standard

© Cancer Research Campaign 1998

dose of epirubicin been given). The variability in AUC, expressed as CV%, was the same for the observed AUC and the predicted AUC. The accuracy of the predicted AUC was confirmed by MPE values of 1.1 (at 120 mg $m^{-2}$), 1.1 (at 90 mg $m^{-2}$) and 1.2 (at 75 mg $m^{-2}$). Precision in the predicted AUC was demonstrated by the MAPE values of 8.6 (at 120 mg $m^{-2}$), 8.0 (at 90 mg $m^{-2}$) and 9.8 (at 75 mg $m^{-2}$).

### Impact of surface area dose adjustment on pharmacodynamics (myelosuppression)

Nadir blood counts were available for 11 of the 32 patients and are shown below. There was a strong correlation between epirubicin AUC and the observed neutrophil nadir ($r = 0.85$, $P < 0.001$). This was reflected in the significantly higher AUC of the five patients with grade 3/4 neutropenia compared with the six with less severe myelosupression (4363 and 1611 ng $ml^{-1}$ h respectively, $P = 0.01$ Mann–Whitney test).

The relative importance of treatment (epirubicin dose), pharmacokinetic (AUC) and patient characteristics (age, height, body weight, % IBW and pretreatment blood counts) in determining leucopenia and neutropenia was investigated using multiple regression analysis. Epirubicin AUC had the strongest relationship with both the absolute neutrophil nadir ($r^2 = 0.72$) and total WBC nadir ($r^2 = 0.63$). This relationship was stronger than that of epirubicin dose with neutrophil nadir or WBC nadir ($r^2 = 0.51$ and 0.47 respectively). Similarly, epirubicin AUC was the only parameter strongly associated with the surviving fraction of both neutrophils and total WBC ($r^2 = 0.62$ and 0.57 respectively).

The potential clinical impact of administering standard doses of epirubicin was explored by estimating the neutropenia that each patient would have experienced had this strategy been employed. The predicted AUC for administration of a standard dose of epirubicin had been calculated above for the 11 patients for whom nadir counts were available. All received single agent epirubicin 75–120 mg $m^{-2}$. Given the linear relationship between AUC and neutrophil nadir described above, the nadir that each patient would have experienced had they been exposed to the predicted AUC was calculated as:

$$\text{Predicted neutrophil nadir} = \frac{\text{observed neutrophil nadir} \times \text{observed AUC}}{\text{Predicted AUC}}$$

Table 4 shows the observed neutrophil nadir (resulting from the mg $m^{-2}$ dose of epirubicin administered) and the predicted AUC (that would be expected had an unadjusted dose of epirubicin been given). The median observed and predicted nadirs were 1.07 and $0.96 \times 10^9$ $l^{-1}$ respectively. There was also no difference in the variability of the observed and predicted nadirs, both having CVs of 71%. The relationship between the observed and predicted neutrophil nadirs is shown in Figure 2. The accuracy and precision of the predicted neutrophil count was confirmed by an MPE and MAPE of 4.5% and 11.6 respectively.

### DISCUSSION

The most important finding in this paper is that there is no relationship between total plasma clearance of epirubicin and any clinical or biochemical parameter or physical characteristic, including body surface area. The implication of these data is that abandoning surface area normalization of epirubicin would not increase the variability in AUC, nor would it significantly affect neutropenia, a measure of epirubicin pharmacodynamics.

These data support the smaller study by Cosolo et al (1994) who also reported no relationship between epirubicin clearance and surface area, although there did appear to be a correlation with lean body mass. Taken with work by Grochow et al (1990) these pharmacokinetic data cast serious doubts on the aptness of normalizing doses according to surface area. It is, however, pharmacodynamic parameters (response or toxicity) that are important in clinical practice. In the current study neutropenia was correlated with epirubicin AUC. Indeed, in a multivariate analysis AUC predicted the neutrophil nadir more strongly than dose of epirubicin or any physical or physiological variable. Other studies have also shown that myelosuppression is influenced by the AUC of epirubicin (Jakobsen et al, 1991), doxorubicin (Ackland et al, 1989) and iododoxorubicin (Robert et al, 1992; Twelves et al, 1994). Therefore, as surface area normalization does not affect epirubicin AUC, it may also do little to reduce interpatient variability in treatment outcome. We investigated this further by studying the effect of abandoning surface area normalization on neutropenia. This analysis suggested that abandoning surface area dose normalization would not significantly alter the pattern of myelosuppression.

These findings have potentially important implications, although they should be viewed with some caution as the patients had a relatively narrow range of surface areas, albeit a range that includes most patients who receive chemotherapy. The first question is whether there are good reasons to continue normalization of epirubicin dose for surface area. Dosing considerations vary in different clinical settings. In situations in which epirubicin is given with curative intent, and dose intensity may be perceived as of paramount importance, clinicians may be especially reluctant to abandon surface area normalization. Epirubicin is, however, often given as palliative treatment when modest differences in dose intensity are unlikely to be significant. Indeed, there are important potential advantages to abandoning routine use of surface area adjustment. Firstly, it would reduce drug wastage and may increase the safety of prescription. Secondly, in routine clinical practice surface area normalization creates the illusion that accurate dose modifications are made for each individual patient. This may deter clinicians from making dose modifications according to other factors such as the tolerability of earlier cycles of treatment. The major possible drawback of forsaking surface area adjustment is that it may lead to overtreatment of 'small' patients and undertreatment of 'large' patients. There is little evidence to support this. Indeed, very large patients are often treated at doses modified for their ideal (rather than actual body) weight or have their surface area 'rounded down' to 2.0 $m^2$.

There are potentially more important implications of ending routine surface area normalization in the area of new drug development. It is certainly appropriate to use surface area normalization to calculate the starting dose for phase I studies as this is extrapolated from animal toxicology data. However, for individual patients it may be more rational not to adjust dose according to surface area. The primary aims of phase I studies include identification of the maximum tolerated dose and proposed phase II dose for a new agent. By assuming that this should be adjusted for surface area we may not fully accomplish that aim. Similarly, phase I trials also aim to determine the causes of pharmacokinetic and pharmacodynamic variability. The importance of physical

© Cancer Research Campaign 1998

characteristics may best be assessed by first using unadjusted drug doses, as for biochemical and haematological parameters, provided the patients' surface area fell within 'normal limits'. As data on pharmacokinetics and toxicity are collected the determinants of variability should be studied systematically, and, when appropriate, incorporated at higher dose levels in the phase I study. These data could then be confirmed by population pharmacokinetic studies in phase II trials.

The current study has shown that there is wide variability in epirubicin pharmacokinetics despite normalization of doses for surface area. Administration of fixed doses of epirubicin would not increase the variability in epirubicin AUC. Likewise, from a clinical perspective, it appears that fixed dosing would not significantly alter the pattern of myelosuppression. There is a need to evaluate systematically the sources of variability in pharmacokinetics and pharmacodynamics in order to develop rational dose recommendations. In some cases this will involve surface area normalization but in others alternate dose strategies may be identified. These considerations apply to drugs in clinical use but are maybe even more relevant for agents under development.

### ACKNOWLEDGEMENTS

We thank Professors RD Rubens and MA Richards under whose care the patients were studied. NAD was supported in part by a grant from Pharmacia.

### REFERENCES

Ackland SP, Ratain MJ and Vozelgang NJ (1989) Pharmacokinetics and pharmacodynamics of long-term continuous infusion doxorubicin. *Clin Pharmacol and Ther* **45**: 340–347

Allerheiligen S, Johnson R, Hatcher B, Freeman P, Tarassof MV and Dorr A (1994) Gemcitabine pharmacokinetics are influenced by gender, body surface area (BSA), and duration of infusion. *Proc Am Soc Clin Oncol* **13**: 339

Bruno R, Hille D, Thomas L et al (1995) Population pharmacokinetics/pharmacodynamics (PK/PD) of docetaxel (Taxotere) in phase II studies. *Proc Am Soc Clin Oncol* **14**: 457

Cosolo W, Morgan DJ, Seeman E, Zimet AS, Mckendrick JJ and Zalcberg JR (1994) Lean body mass, body surface area and epirubicin kinetics. *Anti-Cancer Drugs* **5**: 293–297

Dobbs NA and Twelves CJ (1991) Measurement of epirubicin and its metabolites by high-performance liquid chromatography using an advanced sample processor. *J Chrom Biomed Appl* **572**: 37–42

Dobbs NA, Twelves CJ, Gillies H, James CA, Harper PG and Rubens RD (1995*a*). Gender affects doxorubicin pharmacokinetics in patients with normal liver biochemistry. *Cancer Chemother Pharmacol* **36**: 473–476

Dobbs NA, Twelves CJ, Cruikshank C, Curnow A, Gregory W, Richards MA and Rubens RD (1995*b*). Adaptive dosing for epirubicin (Epi): prospective pharmacokinetic evaluation of nomogram based on serum aspartate aminotransferase (AST). *Proc Am Soc Clin Oncol* **14**: 366

Grochow LB, Baraldi C and Noe D (1990) Is dose normalization to weight or body surface area useful in adults? *J Natl Cancer Inst* **82**: 323–325

Johnson A and Woollard RC (1983) Stripe: an interactive computer programme for the analysis of drug pharmacokinetics. *J Pharmacol Methods* **9**: 193–200

Lotts RS and Hayton WL (1980) Estimation of creatinine clearance from serum creatinine concentration. *Drug Intel Clin Pharm* **12**: 195–201

Reilly J and Workman P (1994) Is body composition an important variable in the pharmacokinetics of anticancer drugs? *Cancer Chemother Pharmacol* **34**: 3–13

Robert J and Hoerni B (1983) Age dependence of the early-phase pharmacokinetics of doxorubicin. *Cancer Res* **43**: 4467–4469

Robert J, Armand JP, Huet S, Kilink-Alaki M, Recondo G and Hurteloup P (1992). Pharmacokinetics and metabolism of 4′-iodo-4′-deoxydoxorubicin. *Br J Cancer* **10**: 1183–1190

Twelves CJ, Dobbs NA, Michael Y, Summers LA, Gregory W, Harper PG, Rubens RD and Richards MA (1992) Clinical pharmacokinetics of epirubicin: the importance of abnormal liver biochemistry tests. *Br J Cancer* **66**: 765–769

Twelves CJ, Dobbs NA, Lawrence MA, Ramirez AJ, Summerhayes M, Richards MA, Towlson KE, Rubens RD (1994) Iododoxorubicin in advanced breast cancer: a phase II evaluation of clinical activity, pharmacology and quality of life. *Br J Cancer* **69**: 726–731

Wade JR, Kelman AW, Kerr DJ, Robert J and Whiting B (1992) Variability in the pharmacokinetics of epirubicin: a population analysis. *Cancer Chemother Pharmacol* **29**: 391

WHO (1979) *Handbook for Reporting Results of Cancer Treatment*. World Health Organization: Geneva

© Cancer Research Campaign 1998